# Risdiplam treatment has not led to retinal toxicity in patients with spinal muscular atrophy

**DOI:** 10.1002/acn3.51239

**Published:** 2020-11-24

**Authors:** Robert C. Sergott, Giulia M. Amorelli, Giovanni Baranello, Emmanuel Barreau, Shannon Beres, Steven Kane, Eugenio Mercuri, Lorenzo Orazi, Melissa SantaMaria, Gemma Tremolada, Diletta Santarsiero, Agnieszka Waskowska, Shigeko Yashiro, Nora Denk, Sabine Fürst‐Recktenwald, Marianne Gerber, Ksenija Gorni, Birgit Jaber, Bjoern Jacobsen, Lutz Mueller, Stephane Nave, Renata S. Scalco, Stefania B. Marzoli, the FIREFISH, SUNFISH, JEWELFISH Working Groups

**Affiliations:** ^1^ Department of Neuro‐Ophthalmology Wills Eye Hospital Philadelphia USA; ^2^ Annesley EyeBrain Center Thomas Jefferson University Philadelphia USA; ^3^ Paediatric Neurology and Nemo Center Catholic University and Policlinico Gemelli Rome Italy; ^4^ The Dubowitz Neuromuscular Centre NIHR Great Ormond Street Hospital Biomedical Research Centre Great Ormond Street Institute of Child Health University College London & Great Ormond Street Hospital Trust London UK; ^5^ Developmental Neurology Unit Fondazione IRCCS Istituto Neurologico Carlo Besta Milan Italy; ^6^ Institute of Myology Paris France; ^7^ Department of Neurology Department of Ophthalmology Stanford University Palo Alto California USA; ^8^ Columbia University Medical Center New York USA; ^9^ Neuro‐Ophthalmology Center Ophthalmology Department IRCCS Istituto Auxologico Italiano Milan Italy; ^10^ Department of Developmental Neurology Medical University of Gdańsk Gdańsk Poland; ^11^ Department of Ophthalmology National Center for Global Health and Medicine (NCGM) Tokyo Japan; ^12^ Roche Pharma Research and Early Development Pharmaceutical Sciences Roche Innovation Center Basel, F. Hoffmann‐La Roche Ltd Basel Switzerland; ^13^ Pharma Development Neurology F. Hoffmann‐La Roche Ltd Basel Switzerland; ^14^ Pharma Development Safety F. Hoffmann‐La Roche Ltd Basel Switzerland; ^15^ PDMA Neuroscience and Rare Disease F. Hoffmann‐La Roche Ltd Basel Switzerland

## Abstract

**Objective:**

Evaluation of ophthalmologic safety with focus on retinal safety in patients with spinal muscular atrophy (SMA) treated with risdiplam (EVRYSDI®), a survival of motor neuron 2 splicing modifier associated with retinal toxicity in monkeys. Risdiplam was approved recently for the treatment of patients with SMA, aged ≥ 2 months in the United States, and is currently under Health Authority review in the EU.

**Methods:**

Subjects included patients with SMA aged 2 months–60 years enrolled in the FIREFISH, SUNFISH, and JEWELFISH clinical trials for risdiplam. Ophthalmologic assessments, including functional assessments (age‐appropriate visual acuity and visual field) and imaging (spectral domain optical coherence tomography [SD‐OCT], fundus photography, and fundus autofluorescence [FAF]), were conducted at baseline and every 2–6 months depending on study and assessment. SD‐OCT, FAF, fundus photography, and threshold perimetry were evaluated by an independent, masked reading center. Adverse events (AEs) were reported throughout the study.

**Results:**

A total of 245 patients receiving risdiplam were assessed. Comprehensive, high‐quality, ophthalmologic monitoring assessing retinal structure and visual function showed no retinal structural or functional changes. In the youngest patients, SD‐OCT findings of normal retinal maturation were observed. AEs involving eye disorders were not suggestive of risdiplam‐induced toxicity and resolved with ongoing treatment.

**Interpretation:**

Extensive ophthalmologic monitoring conducted in studies in patients with SMA confirmed that risdiplam does not induce ophthalmologic toxicity in pediatric or adult patients with SMA at the therapeutic dose. These results suggest that safety ophthalmologic monitoring is not needed in patients receiving risdiplam, as also reflected in the United States Prescribing Information for risdiplam.

## Introduction

### Overview of SMA

Spinal muscular atrophy (SMA) is an autosomal recessive neuromuscular disorder. The most common form is caused by a homozygous deletion or mutation of the survival of motor neuron 1 (*SMN1*) gene on chromosome 5q, which encodes SMN,^1^, ^2^ an essential protein for normal development and functional homeostasis.^3^ Most individuals carry a second gene, *SMN2*, that produces small amounts of functional SMN protein.^4^ SMA is characterized by the progressive loss of spinal motor neurons leading to muscle weakness.^2^ Without therapeutic intervention, it is the leading genetic cause of mortality in infants and young children, with an incidence of 1 in 10,000 live births.^5^

SMA manifests in various degrees of severity defined by age of onset and highest motor milestone achieved;^2^ there are three main subtypes: Type 1 (patients never sit independently), Type 2 (patients can sit but not walk), and Type 3 (patients achieve independent walking).^6^ All subtypes have common clinical signs, including hypotonia, muscle weakness and atrophy, and impaired mobility.^7^

The first two therapies that received Health Authority (HA) approval for the treatment of SMA were as follows: the intrathecally administered *SMN2*‐targeting antisense oligonucleotide nusinersen (SPINRAZA®)^8^ and onasemnogene abeparvovec‐xioi (ZOLGENSMA®), a gene‐transfer therapy that uses a nonreplicating adeno‐associated virus to deliver a functional copy of an *SMN1* gene by a single intravenous infusion in patients aged < 2 years.^9^ Despite these available treatment options, an unmet medical need remains for this broad patient population. For some patients treated with nusinersen, repeated intrathecal administration is either not feasible due to severe scoliosis or too burdensome for both patients and healthcare systems.^10^ Onasemnogene abeparvovec administration requires preparation and administration in a protected environment, systemic glucocorticoid treatments, and close monitoring of liver function and cardiac parameters.^9^ In addition, there is a lack of data in older patients or those with different disease types.

### Overview of risdiplam

Risdiplam (EVRYSDI®) is a centrally and peripherally distributed, oral *SMN2* pre‐mRNA splicing modifier,^11^ which has recently been approved by the FDA for the treatment of patients with SMA aged 2 months and older in the United States^12^ and is currently under HA review in the EU. Risdiplam directly targets the underlying SMA pathophysiology by promoting the inclusion of exon 7 into *SMN2* pre‐mRNA, to generate full‐length *SMN2* mRNA. This molecule increases the production of functional SMN protein in the central nervous system and throughout the body.^13^ Risdiplam has a favorable safety profile and positive efficacy results in infants, children, and adults, supporting its use in SMA.^14^, ^15^, ^16^

#### Preclinical retinal toxicity

In a study of risdiplam administered at three dose levels in cynomolgus monkeys with 39 weeks of daily treatment, retinal toxicity was observed consisting of peripheral photoreceptor degeneration and microcystoid macular degeneration (MMD) in the central retina after 5 − 6 months of treatment.^11^

Photoreceptor degeneration (most pronounced in the periphery) was observed only at the mid and high doses of risdiplam (with the mid‐dose corresponding to exposures >2‐fold of the mean exposures achieved at the proposed therapeutic dose for risdiplam). While some improvement was noted in the layer integrity/organization of the far periphery, the areas with pronounced degeneration of cells (only seen in animals previously treated with the high dose) did not reverse within the 22‐week post‐treatment phase.

MMD, characterized by microcystoid spaces in the inner nuclear layer (INL), was noted in monkeys treated with the high dose only (approximately four times the exposures achieved at the pivotal approved dose in patients with SMA). MMD was reversible during the recovery phase, as observed with spectral domain optical coherence tomography (SD‐OCT), electroretinography, and histopathology.

Experimental evidence suggested that risdiplam has high melanin‐binding capacity, causing its retention in pigmented retinal cells.^11^ The high concentration of risdiplam in these cells may have led to an impairment of lysosomal/autophagosomal function, thus, affecting recycling of photoreceptors.^11^ However, this finding appears to be monkey specific, as pigmented rats showed similar melanin‐binding potential but no retinal toxicity at even higher doses. Possible long‐term consequences associated with such structural changes in the peripheral retina would be impaired night vision or loss of peripheral vision. MMD‐related impairment of vision was not noted in any of the monkeys – not even in animals with pronounced photoreceptor degeneration or MDD.

**Figure 1:**
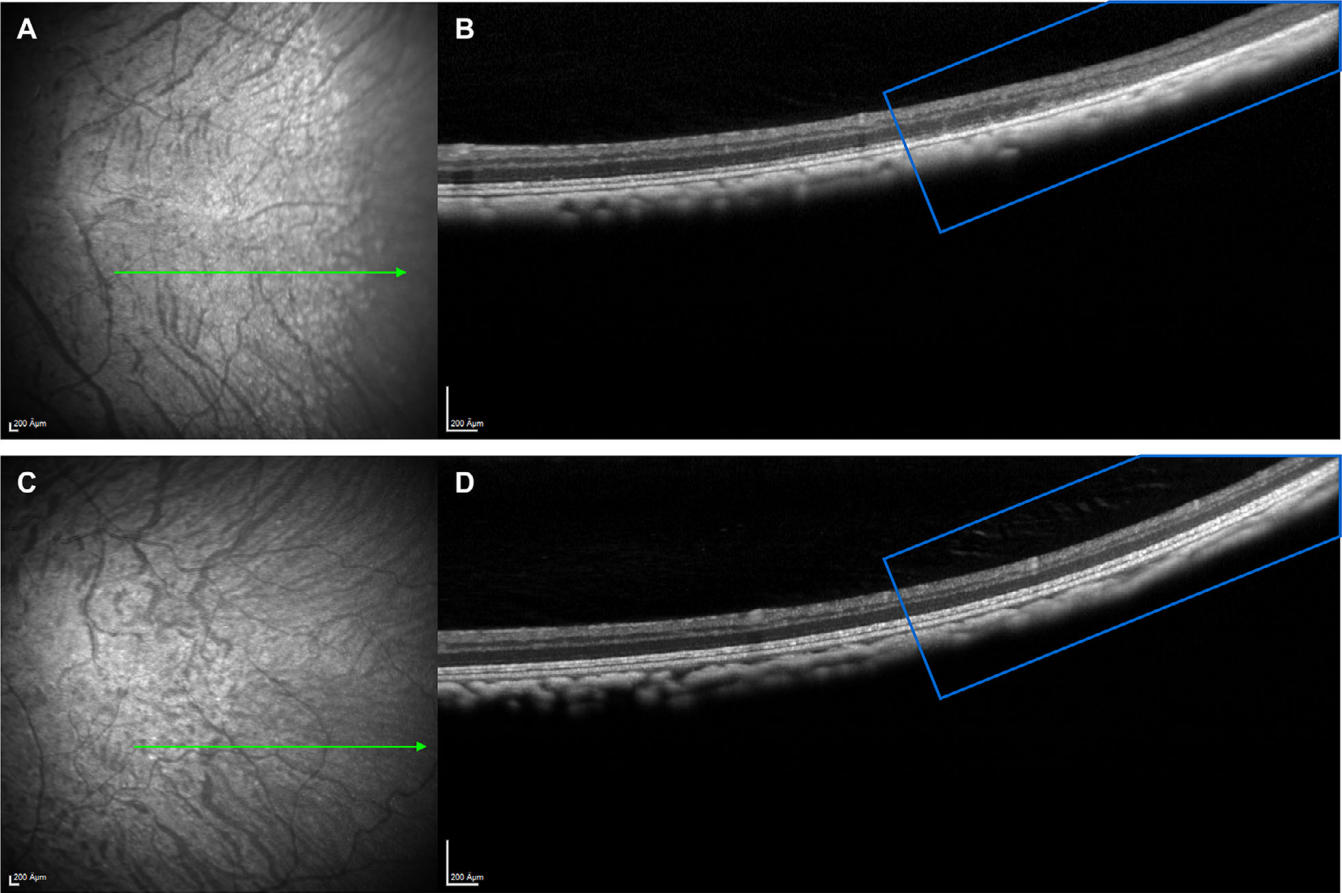
SD‐OCT scan of cynomolgus monkey photoreceptors. (A and B) SD‐OCT scan of photoreceptors from cynomolgus monkey treated with the high dose for 35 weeks. (A) Fundus image: Area with white patches (possibly depigmentation) appears to be on the edge of the area of retinal degeneration. The green arrow indicates the cross‐section location of the OCT scan (1B); (B) OCT scan shows retinal degeneration of the peripheral retina: disorganization, loss of layers and thinning (the retinal layers are visible in the central retina [left side] but disappear in the periphery [right side]). (C and D) For comparison, depicted the fundus image (1C) and OCT (1D) image of an unaffected control cynomolgus monkey. The retinal architecture in the periphery (marked in both OCT images with a blue square) is well preserved in 1D and retinal layers are clearly distinguishable. SD‐OCT, spectral domain‐optical coherence tomography.

Figure 1 depicts an SD‐OCT image from a cynomolgus monkey that demonstrates photoreceptor degeneration, cystic changes in the INL, thinning of the retinal pigment epithelium, and disappearance of the normal ellipsoid and myoid zones of the photoreceptors.

Based on preclinical data, full therapeutic effect was expected to be achieved with a two‐fold increase in SMN protein.^11^ This increase was reached at exposures corresponding to the no‐observed‐adverse‐effect level (NOAEL) for retinal toxicity in monkeys. Risdiplam, therefore, proceeded into clinical development with a dose that corresponds, in terms of systemic exposure in blood, to the exposure at the NOAEL for retinal toxicity in monkeys; in addition, a comprehensive panel of ophthalmologic assessments was included for all studies.

## Methods

### Overview of clinical development studies

**Figure 2:**
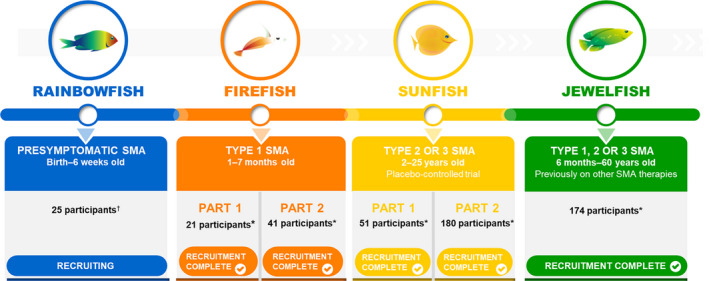
Risdiplam clinical development program overview. *Final participant study numbers; †Target enrollment. SMA, spinal muscular atrophy.

Risdiplam is currently under clinical evaluation in four clinical studies (Fig. 2): a study in infants with symptomatic Type 1 SMA (FIREFISH NCT02913482),^17^ a study in symptomatic, treatment‐naïve patients with Type 2 and 3 SMA (SUNFISH NCT02908685),^18^ a study in symptomatic, non‐naïve patients, (JEWELFISH NCT03032172),^19^ and a study in presymptomatic patients with SMA (RAINBOWFISH NCT03779334).^20^ These studies were designed to assess safety, tolerability, and efficacy of risdiplam.

FIREFISH is a two‐part, multicenter, open‐label study to investigate the safety, tolerability, pharmacokinetics (PK), pharmacodynamics (PD), and efficacy of risdiplam in infants (aged 1 to 7 months at enrollment) with Type 1 SMA.^17^ The study consists of a dose‐finding Part 1 and a confirmatory Part 2 at the dose selected in Part 1. In total, 21 patients were enrolled in Part 1.^14^ Part 2 of FIREFISH has completed recruitment (41 infants) and is ongoing. Following selection of the Part 2 dose, patients in Part 1 entered an extension phase to continue treatment with the Part 2 dose.

SUNFISH is a two‐part, multicenter, randomized, placebo‐controlled, double‐blind study to investigate safety, tolerability, PK/PD, and efficacy of risdiplam in patients with Type 2 and 3 SMA (aged 2–25 years).^18^ The study consists of a dose‐finding Part 1 and a confirmatory Part 2 at the dose selected in Part 1. In total, 51 patients were enrolled in Part 1.^15^ Patients receiving placebo were switched to active treatment at the dose tested in their respective cohort after a minimum 12‐week, placebo‐controlled treatment period. After selection of the dose for Part 2, all Part 1 patients received the pivotal dose during an open‐label extension phase. Part 2 has completed recruitment (180 patients), is ongoing, and was still blinded at the clinical cut‐off date (CCOD) – 06 September 2019.

JEWELFISH is an open‐label, noncomparative study of risdiplam in patients with SMA who were previously enrolled in Roche Study BP29420 (MOONFISH) with the splicing modifier RG7800 (RO6885247) (development discontinued) or previously treated with nusinersen, onasemnogene abeparvovec, or olesoxime (previous Roche acquired development compound, since discontinued).^19^ Forty‐five patients of the planned target of 180 had been enrolled up to the CCOD of 28 June 2019; these 45 patients (43 patients with Type 2 or 3 SMA and two with Type 1 SMA) had previously received nusinersen (*n* = 24), olesoxime (*n* = 12), or RG7800 (*n* = 9). JEWELFISH has completed recruitment (174 patients) and is ongoing.

Risdiplam is approved for treatment of SMA in the United States and is under HA review in EU. The new drug application submitted to the FDA/EMA included extensive safety data, including ophthalmologic safety data in patients with SMA participating in the FIREFISH, SUNFISH, and JEWELFISH studies.

### Study oversight

The FIREFISH, SUNFISH, and JEWELFISH trials were approved by an ethics committee at each study site and were conducted in accordance with Good Clinical Practice guidelines and with the World Medical Association Declaration of Helsinki. Written informed consent was provided by patients or the caregivers of patients.

### Analysis population

In total, 338 patients were included in the analysis (Table 1):

- “*All open‐label/unblinded risdiplam*” pool consisting of patients treated with at least one dose of risdiplam in FIREFISH Parts 1 and 2, SUNFISH Part 1, and JEWELFISH (n = 158).
  - Patients in this pool were further subdivided into two groups by SMA type:
    ▪ patients with Type 1 SMA from FIREFISH Parts 1 and 2 (n = 62) and from JEWELFISH (n = 2)
    ▪ patients with Types 2 and 3 SMA from JEWELFISH (n = 43) and from SUNFISH Part 1 (n = 51).
- “*Blinded Part 2 SUNFISH*” pool with Types 2 and 3 SMA (n = 180) (2:1 randomization risdiplam:placebo), with 120 patients exposed to risdiplam.

**Table 1:** Patient demographic characteristics at baseline.

	*“All open label/unblinded risdiplam*” pool			*“Blinded Part 2 SUNFISH*” pool
	Type 1 SMA (*n* = 64)	Type 2/3 SMA (*n* = 94)	All patients (*n* = 158)	All patients (*n* = 180)
Age at first dose (years)				
Mean (SD)	0.78 (1.86)	15.74 (12.34)	9.68 (12.07)	10.0 (5.9)^1^
Median	0.48	13.76	5.76 0.6–14.4	9.0^1^
IQR	0.4–0.6	6.8–19.3	0.2–60.9	5–14^1^
Min–Max	0.2–12.0	2.8–60.9		2–25^1^
Age group at first dose, n (%)				
0 to < 2 years	62 (96.9)	0	62 (39.2)	0^1^
2 to < 12 years	1 (1.6)	42 (44.7)	43 (27.2)	112 (62.3)^1^
12 to < 18 years	1 (1.6)	27 (28.7)	28 (17.7)	46 (25.6)^1^
>18 years	0	25 (26.6)	25 (15.8)	22 (12.2)^1^
Gender, n (%)				
Male	25 (39.1)	47 (50.0)	72 (45.6)	89 (49.4)
Female	39 (60.9)	47 (50.0)	86 (54.4)	91 (50.6)
Race, *n* (%)				
Asian	18 (28.1)	2 (2.1)	20 (12.7)	35 (19.4)
White	37 (57.8)	84 (89.4)	121 (76.6)	126 (70.0)
Unknown	9 (14.1)	8 (8.5)	17 (10.8)	16 (8.9)
Other^2^	0	0	0	3 (1.7)

IQR, interquartile range; SD, standard deviation; SMA, spinal muscular atrophy.

^1^ Age at screening. Data cut‐off: 28 June 2019.

^2^ Includes Black or African American and Mixed Race.

### Eligibility criteria

Eligibility criteria for the respective studies are listed in the supplementary appendices. Patients with a history of ophthalmologic disease within the last year that may have potentially confounding ophthalmologic baseline findings or patients unable to perform the ophthalmologic assessments were ineligible to participate. Patients with any prior use of chloroquine, hydroxychloroquine, retigabin, vigabatrin, or thioridazine, or use of other medications known to or suspected of causing retinal toxicity within 1 year prior to randomization were also ineligible.

### Ophthalmologic monitoring

Ophthalmologic assessments included:

- ophthalmologic examinations appropriate for age, with at least slit lamp dilated examination with fundus examination and intraocular pressure (IOP) testing performed for all patients
- retinal imaging (fundus autofluorescence [FAF] in adult patients and cooperative children, SD‐OCT using Bioptigen® hand‐held SD‐OCT for infants and young children and Spectralis® (Heidelberg Engineering) for older patients, and fundus photography) to detect structural changes in the retina
- visual function testing included:
  - Best Corrected Visual Acuity (BCVA) and Sloan Low Contrast Visual Acuity (LCVA) testing to detect potential impairment in central vision
  - Visual Field Threshold Perimetry or other age‐appropriate testing to detect potential impairment in peripheral vision.

As studies progressed in the absence of any signals emerging from this monitoring, and in consideration of the extreme burden imposed upon this severely disabled patient population, Sloan LCVA and FAF were no longer performed and the frequency of some assessments was reduced over subsequent protocol amendments. However, the main assessment, SD‐OCT, remained for all patients, at a bi‐monthly schedule in SUNFISH and FIREFISH and changed from bi‐monthly (initially) to every 3 months in JEWELFISH with repeats being systematically requested in case of insufficient quality of images. Table 2 includes details on the ophthalmologic assessments.

**Table 2:** Ophthalmologic assessments in the risdiplam clinical studies

Ophthalmologic assessments	FIREFISH	SUNFISH	JEWELFISH
SD‐OCT	X	X	X
Fundus photography	X	X	X
FAF	–	X	X
Visual field threshold perimetry	–	X	X
Fundus examination	X	X	X
Slit lamp	X	X	X
Intraocular pressure	X	X	X
BCVA	–	X	X
Fix and follow	X	X	X
Sloan low contrast	–	X	X
Simple visual field test	–	X	X

X = assessment/examination is in the study schedule of assessments; – = assessment/examination is not in the study schedule of assessments.

BCVA, Best Corrected Visual Acuity; FAF, fundus autofluorescence; SD‐OCT, spectral domain optical coherence tomography.

**Figure 3:**
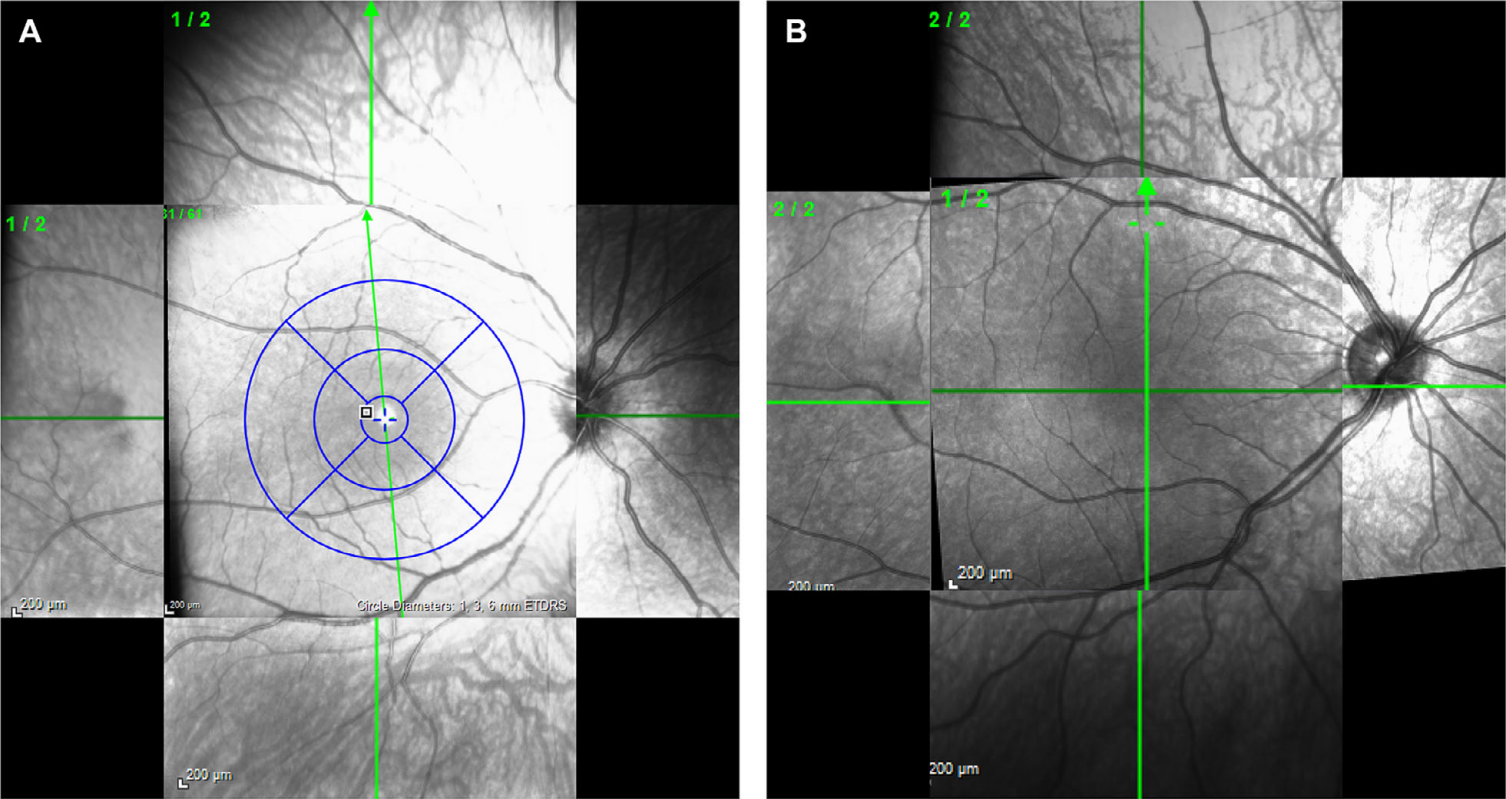
Peripheral cross‐hair posterior pole scan overlay images. (A) Posterior pole with peripheral cross‐hair images. The posterior pole also provides measurement data for the bulls eye area in the window; (B) Foveal cross‐hair and peripheral cross‐hair images.

A dedicated independent central reader, Annesley Eye Brain Center (AEBC; formerly the Optic Nerve Research Center), Thomas Jefferson University (Philadelphia, PA, USA), provided training for the site ophthalmologists and technicians and performed stringent quality reviews of all images. To increase exploration of the peripheral retina, the central reader developed an innovative SD‐OCT protocol that combined posterior pole and cross‐hair scans captured following nasal, temporal, superior, and inferior fixation (Fig. 3).

Changes from baseline that occurred through the assessments, but were no longer observed at the last available assessment, were considered related to test/retest variability or transient findings that disappeared despite ongoing risdiplam treatment. These findings were, therefore, not considered to be risdiplam‐induced effects and are not presented. Only findings present at the patient’s last available assessment are summarized.

Predefined criteria (Table 3) were used to reduce assessment variability and specify which findings should be considered abnormal or potentially clinically significant changes from baseline (thereafter referred to as “findings”). Findings were reviewed by a central reader who provided recommendations for additional assessments in consultation with the site ophthalmologist, if required. For the purpose of data presentation, findings in both eyes at the same assessment or the same finding observed in different ophthalmologic assessments are counted separately.

**Table 3:** Criteria for clinically significant change from baseline (“finding”) in ophthalmologic assessments

Ophthalmologic assessments	Criteria for abnormal or potentially clinically significant results
SD‐OCT	A clinically significant change from baseline (as assessed by an independent central reader, or local ophthalmologist); or an abnormal macula OCT assessment; or a result other than “Not Applicable” for the macula OCT diagnosis
Fundus photography	A clinically significant change from baseline (as assessed by an independent central reader, or local ophthalmologist); or an abnormal photo assessment; or a result other than ‘Not Applicable’ for the photo diagnosis; or a result of ‘Yes’ for pigmentation observed
FAF	A clinically significant change from baseline (as assessed by an independent central reader; or local ophthalmologist) or an abnormal FAF macula assessment; or a result of “Yes’ for hypofluorescence present; or a result of “Yes’ for hyperfluorescence present
Visual field threshold perimetry	A clinically significant change from baseline (as assessed by an independent central reader, or local ophthalmologist); or result other than ‘Normal,’ ‘Unreliable,’ ‘Not Applicable,’ or ‘Not Performed’ in the visual field pattern assessment or a result of ‘Worse’ (or ‘Worse Compared to Unscheduled Baseline’) for the visual field comparison
Fundus examination	A clinically significant change (worse) from baseline (as assessed by the local ophthalmologist); or an abnormal result; or a retinal break; or a retinal detachment
Slit lamp	A clinically significant change (worse) from baseline (as assessed by the local ophthalmologist); or an abnormal result
Visual testing	A clinically significant change (worse) from baseline (as assessed by the local ophthalmologist); or an abnormal result
Intraocular pressure	For digital palpitation method: a clinically significant change (worse) from baseline (as assessed by the local ophthalmologist); or an abnormal result; or for methods other than digital palpitation: Postbaseline intraocular pressure < 10 mmHg or> 25 mmHg; or increase or decrease of> 5 mmHg from baseline
BCVA	A decrease of ≥ 9 optotypes from baseline, an increase of ≥ 0.18 in the early treatment diabetic retinopathy study log score from baseline; or for off‐chart visual acuity: a clinically significant change from baseline
Fix and follow	A clinically significant change (worse) from baseline (as assessed by the local ophthalmologist); or an abnormal result
Sloan low contrast	A decrease of ≥ 7 in the total number of letters correctly read from baseline: or an increase of ≥ 0.14 in the log contrast sensitivity from baseline
Simple visual field test	A clinically significant change (worse) from baseline (as assessed by the local ophthalmologist); or an abnormal result

BCVA, Best Corrected Visual Acuity; FAF, fundus autofluorescence; SD‐OCT, spectral domain optical coherence tomography.

The duration of ophthalmologic monitoring was estimated based on the key assessment SD‐OCT, which was among the most frequently performed assessments (once every 8 or 12 weeks depending on the study). Patient numbers were estimated after discounting exposure in patients on placebo based on 2:1 randomization in SUNFISH Part 2.

### Adverse events (AEs)

AEs were reported from the first dose of study treatment and continuously throughout the observation period. AEs were coded using the Medical Dictionary for Regulatory Activities (MedDRA) Version 22.0. All AEs coding to the primary System Organ Class (SOC) “Eye Disorders” as well as ophthalmologic events coding to other SOCs are presented.

## Results

### Patient population

A total of 338 patients (64 with Type 1 SMA; 274 with Type 2/3 SMA) received at least one dose of the assigned treatment; 278 patients received risdiplam and 60 patients received placebo.

In the “*all open‐label/unblinded risdiplam*” pool, approximately 80% of the exposure time to risdiplam was at the therapeutic dose and 20% at subtherapeutic doses. In the “*blinded Part 2 SUNFISH*” pool, all patients who were treated with risdiplam received the therapeutic dose.

Overall, 13 patients withdrew from the study prior to the CCOD 28 June 2019 (seven patients with Type 1 died due to SMA‐related respiratory complications and six with Types 2/3 SMA withdrew for nonsafety‐related reasons).

### Demographic characteristics

Demographic characteristics (Table 1) are reflective of the inclusion criteria and geographical distribution of study sites. Patients were aged 2.2 months–60.9 years. While the majority were Caucasian, approximately 15% of patients were Asian. The percentage of male and female patients was balanced.

### Duration of ophthalmologic follow‐up

The longest duration of follow‐up was 30.2 months; 91.7% of patients (310 of 338) reached at least the first assessment time point postbaseline and successfully performed the first postbaseline SD‐OCT (Table 4). At the CCOD (28 June 2019), 28 patients had not reached the first assessment time point postbaseline: one patient in FIREFISH Part 1 (who died prior to reaching the first assessment time point), three patients in FIREFISH Part 2, and 24 patients in JEWELFISH. Overall, 85% of all patients successfully completed their SD‐OCT assessments as scheduled.

**Table 4:** SD‐OCT assessments performed at last visit

SD‐OCT	SUNFISH Part 1^1^ (*n* = 51)	SUNFISH Part 2^1^ (*n* = 180)	FIREFISH Part 1 (*n* = 21)	FIREFISH Part 2 (*n* = 41)	JEWELFISH (*n* = 45)	All patients (*N* = 338)
Patients with at least one postbaseline visit, n (%)	51 (100)	180 (100)	20 (95.2)	38 (92.7)	21 (46.7)	310 (91.7)

CCOD, clinical cut‐off date; SD‐OCT, spectral domain optical coherence tomography.

^1^ Patients in SUNFISH were randomized 2:1 to receive risdiplam or placebo, respectively. All available dosing information up to the last site visit prior to the CCOD is included. Baseline is the last measurement prior to the patients first dose of study medication, either placebo or risdiplam. CCOD: 28 June 2019.

In total, 245 patients treated with risdiplam had successful postbaseline SD‐OCT monitoring for at least 2 months. Fifteen patients had SD‐OCT assessments for 2.5 years, 52 patients for 2 years, 143 patients for 1 year, and 233 patients for 6 months.

### Ophthalmologic safety findings

Overall, 207 patients had 477 ophthalmologic safety findings at the last ophthalmology visit. Most findings were either changes in retinal layer thickness due to angulation variability during SD‐OCT assessment, measurement variability in IOP, BCVA, SLOAN LCVA, or threshold perimetry, which were not clinically significant. Findings that were deemed clinically significant and reported as AEs are described below.

### Ophthalmologic AEs

Overall, in the “*all open‐label/unblinded risdiplam*” pool (*n* = 158), nine patients (5.7%) had an ophthalmologic AE coding to SOC Eye Disorders (Table 5). No ophthalmologic events were reported in other SOCs. Events were mild to moderate in intensity, were reported as unrelated to risdiplam, resolved despite ongoing treatment, and were not suggestive of risdiplam‐induced effects. All ophthalmologic AEs were mono‐ocular (no events involved both eyes).

**Table 5:** Ophthalmologic AEs in the “*all open‐label/unblinded risdiplam*” pool

Ophthalmologic AEs	Type 1 SMA (*n* = 64)	Type 2/3 SMA (*n* = 94)	All patients (*N* = 158)
Total number of patients with at least one AE, *n* (%)	3 (4.7)	6 (6.4)	9 (5.7)
Overall total number of events, *n*	4	7	11
Eye disorders, *n* (%)			
Conjunctival hyperemia	2 (3.1)	0	2 (1.3)
Blepharitis	0	1 (1.1)	1 (0.6)
Dry eye	0	1 (1.1)	1 (0.6)
Eczema eyelids	0	1 (1.1)	1 (0.6)
Eye allergy	0	1 (1.1)	1 (0.6)
Macular cyst	1 (1.6)	0	1 (0.6)
Ocular hyperemia	0	1 (1.1)	1 (0.6)
Photopsia	0	1 (1.1)	1 (0.6)
Retinal exudates	1 (1.6)	0	1 (0.6)
Vision blurred	0	1 (1.1)	1 (0.6)

Investigator text for AEs is coded using MedDRA version 22.0. Percentages are based on the n number in the column headings. For frequency counts by preferred term, multiple occurrences of the same AE in an individual are counted only once. For frequency counts of “total number of events” rows, multiple occurrences of the same AE in an individual are counted separately. Includes AEs with onset from first dose of risdiplam up to the CCOD, 28 June 2019.

AE, adverse event; CCOD, clinical cut‐off date; MedDRA, Medical Dictionary for Regulatory Activities; SMA, spinal muscular atrophy.

**Figure 4:**
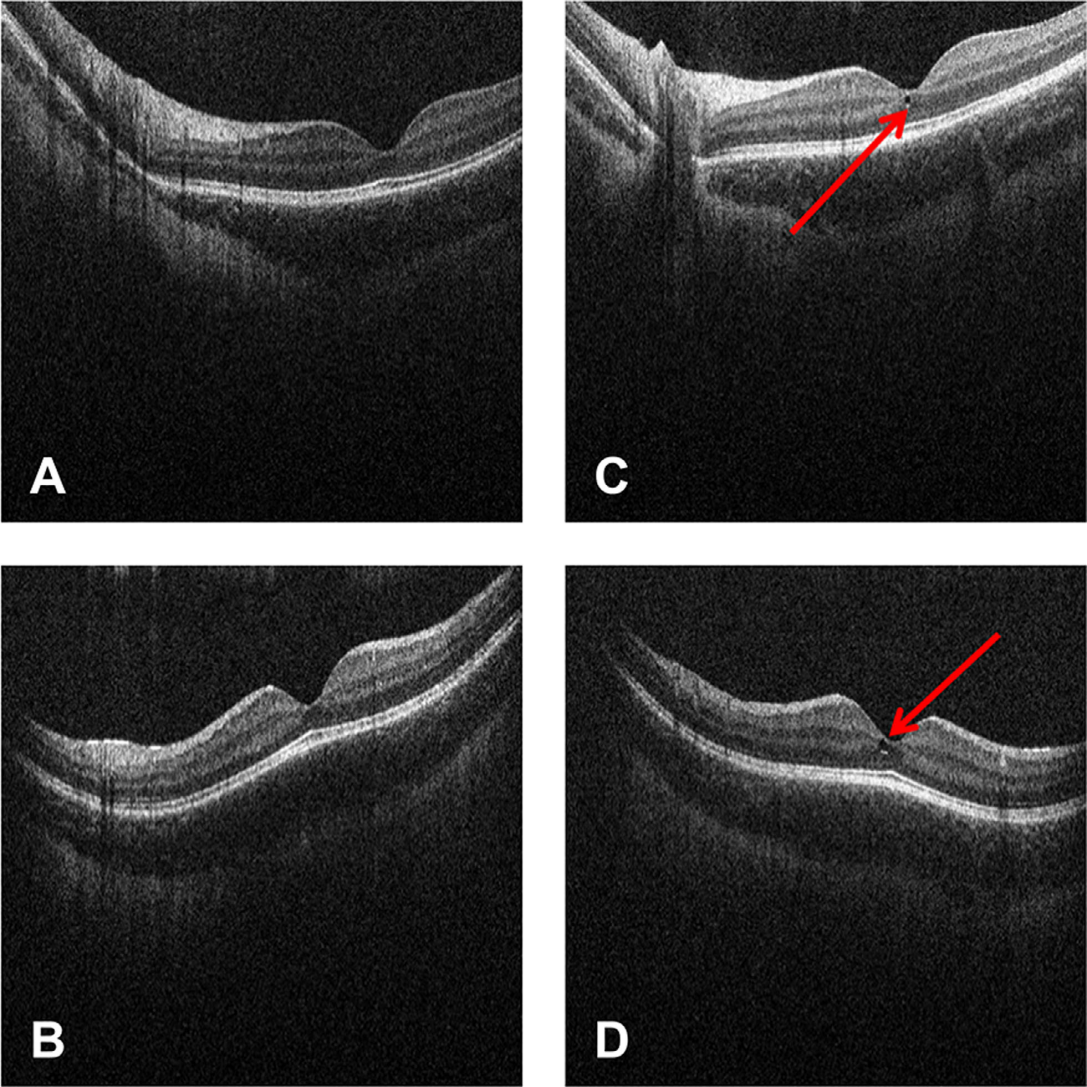
SD‐OCT scan of foveal microcyst in patient with SMA. SD‐OCT scans (left eye) from patient from all the patients with SMA pool at screening and the Week 8 visit. (A) Horizontal scan at screening visit; (B) Vertical scan at screening visit; (C) Horizontal scan at Week 8 visit; (D) Vertical scan at Week 8 visit. Red arrows indicate foveal microcyst. SD‐OCT, spectral domain optical coherence tomography; SMA, spinal muscular atrophy.

Three of these events were reported by the study ophthalmologist as a result of the scheduled ophthalmologic assessment and resolved despite ongoing treatment with risdiplam:

- macular cyst (foveal microcyst) in left eye at Week 8 SD‐OCT; no longer present at an unscheduled SD‐OCT 4 weeks later and consistent with retinal maturation (Fig. 4A–D)
- small isolated retinal exudates in left eye on fundus photographs; resolved at an unscheduled assessment 8 weeks later
- conjunctival hyperemia associated with acute blepharitis, diagnosed at ocular examination by the study ophthalmologist at Week 43; resolved within 6 days under treatment with tobramycin eye drops.

In the “*blinded Part 2 SUNFISH*” pool (treatment arm unknown), 13 (7.2%) patients had ophthalmologic AEs coding to SOC Eye Disorders (Table 6), which were mild to moderate in intensity and resolved with the exception of two incidences of subcapsular cataract and one of mild visual impairment. The latter occurred in a 5‐year‐old patient, who had an event of visual impairment without corresponding findings at ophthalmologic assessments, which had been observed in the context of headache; however, this finding could not be verified or validated by two certified board ophthalmologists at the reading center. The two events of subcapsular cataract were observed during ophthalmologic examination in a 13‐year‐old Asian female who had a subcapsular cataract (left eye) and posterior capsule opacification (right eye); however, these findings also could not be verified or validated by a certified board of ophthalmologists and they were not confirmed by red reflex images acquired after the CCOD. In addition, one patient had a small conjunctival nevus at slit lamp examination (right eye) at Week 17 that was stable through to Week 43 and, therefore, may have been missed at screening. This finding was reported as an AE of mild, unrelated, and unresolved eye nevus.

**Table 6:** Ophthalmologic AEs in the “*Blinded Part 2 SUNFISH*” pool

Ophthalmologic AEs	*“Blinded Part 2 SUNFISH”* pool All patients (n = 180)
Total number of patients with at least one AE, n (%)	13 (7.2)
Overall total number of events, n	16
Eye disorder AEs, *n* (%)	
Dry eye	3 (1.7)
Conjunctivitis allergic	2 (1.1)
Eye pain	2 (1.1)
Cataract subcapsular	1 (0.6)
Conjunctival hemorrhage	1 (0.6)
Eye pruritus	1 (0.6)
Eyelid disorder	1 (0.6)
Lacrimation increased	1 (0.6)
Ocular hyperemia	1 (0.6)
Posterior capsule opacification	1 (0.6)
Vision blurred	1 (0.6)
Visual impairment	1 (0.6)

Investigator text for AEs is coded using MedDRA version 22.0. Percentages are based on the n number in the column headings. For frequency counts by preferred term, multiple occurrences of the same AE in an individual are counted only once. For frequency counts of “total number of events” rows, multiple occurrences of the same AE in an individual are counted separately. Patients in SUNFISH were randomized 2:1 to receive risdiplam or placebo, respectively. Includes AEs with onset from first dose of risdiplam up to the CCOD, 28 June 2019.

AE, adverse event; CCOD, clinical cut‐off date; MedDRA, Medical Dictionary for Regulatory Activities.

## Discussion

Extensive ophthalmologic monitoring assessing retinal structure and visual function in three prospective clinical studies, monitored by a dedicated independent central reader, demonstrated that risdiplam did not induce ophthalmologic toxicity in pediatric and adult patients with SMA. The studies included patients aged 2 months to 60 years at enrollment, so some patients were infants whose retinas had not yet fully matured.^21^ AEs observed in the studies were not suggestive of risdiplam‐induced toxicity (identified in preclinical evaluation of primates) and resolved with ongoing treatment. No ophthalmic‐related AEs led to study withdrawal in any patient receiving risdiplam.

The preclinical toxicity observed in non‐human primates^11^ at exposures substantially greater than the NOAEL was detected in the retina periphery extending into the central part with MMD only at the highest dose. The observed toxicity in non‐human primates was suggested to be related to an impaired lysosomal function of the retinal pigment epithelium. These preclinical toxicity findings were not found in any patient receiving risdiplam in any clinical study. All subjects maintained clinically normal visually oriented behavior throughout the study, without any reports of decreased central acuity or peripheral visual field, as assessed by the subjects’ reports when possible and/or by the reports of parents and caregivers.

In this study, although melanin content of the retinal pigment epithelium shows only little racial variation (unlike choroidal melanocytes),^22^ no increased risk of toxicity was observed in Asian patients (approximately 15% of the study population) who might potentially have exhibited higher melanin binding.

The comprehensive set of ophthalmologic assessments, which included imaging and visual function testing, was tailored to evaluate all aspects of ophthalmologic safety, with a special focus on retinal structure using SD‐OCT. The central reader developed an innovative SD‐OCT protocol scan that was captured following nasal, temporal, superior, and inferior fixation when possible to enhance even further the exploration of the peripheral retina. In addition, no functional impairment was observed in visual field assessments using threshold perimetry in patients able to maintain prolonged fixation despite their significant disability.

Although patients with SMA have significant physical disability,^2^ 85% of all patients performed all scheduled assessments. This demonstrates the strong collaboration from the patients and their caregivers, as well as site ophthalmologists, technicians, and study staff at specialized centers who made a substantial effort to adapt the assessments to the patients’ physical conditions. Multiple repeat testing was required to capture images of high quality for standardized longitudinal evaluations.

The high number of ophthalmologic safety findings observed compared with baseline was expected based on variability in OCT image angles due to subjects’ head and neck weakness. No patients treated with risdiplam at the recommended dose (n = 245) had a finding that indicated risdiplam‐induced toxicity at their last assessment. AEs were also not suggestive of ophthalmologic toxicity. The clinical trial framework was essential in enabling reliable ophthalmologic evaluation.

Extensive ophthalmologic monitoring conducted in studies in patients with SMA confirmed that risdiplam does not induce ophthalmologic toxicity in pediatric and adult patients with SMA at the therapeutic dose. These results suggest that safety ophthalmologic monitoring is not needed in patients receiving risdiplam, as also reflected in the recently published United States Prescribing Information for risdiplam.^12^ The study sponsor is monitoring longer‐term ophthalmologic safety of risdiplam in the open‐label extension parts of the ongoing clinical studies.

## Conflicts of Interest

RS received fees from Annesley EyeBrain Center at Jefferson during the conduct of the study; GB has received fees from F. Hoffmann‐La Roche during the conduct of the study and personal fees from F. Hoffmann‐La Roche, outside the submitted work; EB reports grants and nonfinancial support from Hofmann La Roche, during the conduct of the study; EM has received fees from F. Hoffmann‐La Roche for advisory boards and is a principle investigator for the Roche studies; ND, SFR, BJ, and SN are employees of F. Hoffmann‐La Roche; MG, KG, BiJ, LM, and RS are employees of, and hold shares in, F. Hoffmann‐La Roche; GMA, SBe, SK, LO, MSM, GT, DS, AW, SY, and SBM have no conflicts of interest.

## Author Contributions

All authors contributed to the study conception and design. Analysis and interpretation were performed by all authors. The first draft of the manuscript was written by RS and MG. All authors commented on previous versions of the manuscript. All authors read and approved the final manuscript.

## Supporting information


**Appendix S1.** (A) Study oversight (complete lists of the FIREFISH, SUNFISH and JEWELFISH Working Groups). (B) Eligibility criteria for the FIREFISH study. (C) Eligibility criteria for the SUNFISH study. (D) Eligibility criteria for the JEWELFISH study.
