# Supplementary material for: Risdiplam treatment has not led to retinal toxicity in patients with spinal muscular atrophy
Source: Ann Clin Transl Neurol. 2020 Nov 24;8(1):54–65. doi: 10.1002/acn3.51239 (PMC7818230; doi:10.1002/acn3.51239)
Supplement: Supplementary file 1 — Appendix S1. (A) Study oversight (complete lists of the FIREFISH, SUNFISH and JEWELFISH Working Groups). (B) Eligibility criteria for the FIREFISH study. (C) Eligibility criteria for the SUNFISH study. (D) Eligibility criteria for the JEWELFISH study. [file ACN3-8-54-s001.docx]

**SUPPLEMENTARY APPENDIX S1.**

Supplement to: Sergott RC, Amorelli GM, Baranello G. et al. Risdiplam treatment has not led to retinal toxicity in patients with spinal muscular atrophy

Contents

[S1A. STUDY OVERSIGHT 3](#_Toc53759956)

[FIREFISH Working Group 3](#_Toc53759957)

[SUNFISH Working Group 4](#_Toc53759958)

[JEWELFISH Working Group 8](#_Toc53759959)

[S1B. FIREFISH ELIGIBILITY CRITERIA 10](#_Toc53759960)

[Inclusion criteria 10](#_Toc53759961)

[Exclusion criteria 11](#_Toc53759962)

[S1C. SUNFISH ELIGIBILITY CRITERIA 14](#_Toc53759963)

[Inclusion criteria 14](#_Toc53759964)

[Exclusion criteria 16](#_Toc53759965)

[S1D. JEWELFISH ELIGIBILITY CRITERIA 18](#_Toc53759966)

[Inclusion criteria 18](#_Toc53759967)

[Exclusion criteria 21](#_Toc53759968)

#

# S1A. STUDY OVERSIGHT

## FIREFISH Working Group

**Principal investigators (in bold) and site study personnel**

*Belgium*: **Nicolas Deconinck, M.D.,** Ophthalmologists: Irina Balikova M.D. Patricia Delbeke M.D., Inge Joniau M.D.; Physiotherapists: Valentine Tahon, Sylvia Wittevrongel; Study coordinator: Elke De Vos; *Brazil*: **Edmar Zanoteli M.D.,** Rodrigo de Holanda Mendonça, M.D., Ciro Matsui Jr, M.D.; Ophthalmologists*:* Ana Letícia Fornazieri Darcie, Cleide Machado, Maria Kiyoko Oyamada, Daniel de Souza Costa; Physiotherapists: Joyce Martini, Graziela Polido, Juliana Rodrigues Iannicelli; *China*: **Yi Wang, M.D.,** Chaoping Hu M.D., Yiyun Shi M.D., Shuizhen Zhou M.D., Xiaomei Zhu M.D.; Ophthalmologists: Chen Qian, Li Shen, Ying Xiao, Zhenxuan Zhou; Physiotherapists: Hui Li, Sujuan Wang; **Hui Xiong, M.D.,** Tian Sang M.D., Cuijie Wei M.D., Jing Wen M.D.; Ophthalmologists: Yiwen Cao, Jing Wen; Physiotherapists: Wenzhu Li, Lun Qin; *Croatia*: **Nina Barisic, M.D.,** Ophthalmologists: Ivan Celovec, Martina Galiot Delic, Petra Kristina Ivkić, Nenad Vukojević; Physiotherapists: Ivana Kern, Boris Najdanovic, Marin Skugor; *France*: **Laurent Servais, M.D., Odile Boespflug-Tanguy, M.D.,** Teresa Gidaro M.D., Andrea Seferian M.D.; Ophthalmologists: Emmanuel Barreau, Elodie Da Cunha, Céline Lambotin, Nabila Mnafek, Helene Peche; Physiotherapists: Stephanie Gilabert, Allison Grange, Charlotte Lilien, Darko Milascevic, Ariadna Perticari, Shotaro Tachibana; *Italy*: **Giovanni Baranello, M.D., Riccardo Masson, M.D.,** Emanuela Pagliano M.D.; Ophthalmologists: Stefania Bianchi Marzoli, Diletta Santarsiero, Myriam Garcia Sierra, Gemma Tremolada; Physiotherapists: Maria Teresa Arnoldi, Marta Vigano, Riccardo Zanin; **Claudio Bruno, M.D.,** Ophthalmologists: Enrico Priolo, Giuseppe Rao, Enrica Spaletra, Lorenza Sposetti, Elisa Tassara; Physiotherapists: Valentina Lanzillotta, Simone Morando, Paola Tacchetti, Ambra Zuffi; **Giacomo Pietro Comi, M.D.,** Alessandra Govoni M.D.; Ophthalmologists: Silvia Gabriella Osnaghi, Valeria Minorini; Physiotherapists: Francesca Abbati, Federica Fassini, Michaela Foa, Amalia Lopopolo, Elisa Minuti; **Eugenio Mercuri, M.D.,** Marika Pane M.D., Concetta Palermo M.D., Maria Carmela Pera M.D.; Ophthalmologists: Giulia Maria Amorelli, Costanza Barresi, Gugliemo D’Amico, Lorenzo Orazi; Physiotherapists: Giorgia Coratti, Roberto De Sanctis; *Japan*: **Yasuhiro Takeshima, M.D.,** Ophthalmologists: Fumi Gomi, Naoki Kimura, Takanobu Morimatsu, Mana Okamoto; Physiotherapists: Toru Furukawa; *Poland*: **Maria** **Mazurkiewicz-Bełdzińska, M.D.,** Ophthalmologists: Mateusz Koberda, Natalia Kubiak, Urszula Stodolska-Koberda, Agnieszka Waśkowska; Physiotherapists: Jagoda Kolendo, Agnieszka Sobierajska-Rek; *Russia*: **Dmitry Vlodavets, M.D.,** Evgenia Melnik M.D.; Ophthalmologists: Natalya Leppenen, Nataliya Yupatova; Physiotherapists: Elena Litvinova, Anastasya Monakhova, Yulia Papina, Olga Shidlovsckaia; *Switzerland*: **Andrea Klein, M.D.,** Cornelia Enzmann M.D., Elea Galiart M.D.; Ophthalmologists: Konstantin Gugleta, Patricia Siems; Physiotherapists: Verena Kreiliger*,* Christine Wondrusch Haschke; *Turkey*: **Haluk Topaloglu, M.D.,** **Ibrahim Oncel, MD.,** Didem Ardicli M.D., Nesibe Eroglu Ertugrul M.D., Hizal Gharibzadeh M.D., Ceren Gunbey M.D., Bahadir Konuskan M.D, Selen Serel Arslan M.D., Elams Ebru Yalcin M.D. Fatma Gokcem Yildiz Sarikaya M.D.; Ophthalmologists: Bora Eldem, Sibel Kadayıfçılar; Physiotherapists: Ipek Alemdaroglu, Aynur Ayse Karaduman, Oznur Tunca Yilmaz; *United States of America*: **Basil T. Darras, M.D.,** Ophthalmologists: Lucia Ambrosio, Anne Fulton, Anna Maria Baglieri; Physiotherapists: Courtney Dias, Elizabeth Maczek, Elizabeth Mirek, Amy Pasternak; **John W. Day, M.D.,** Ophthalmologist: Shannon Beres; Physiotherapists: Tina Duong, Richard Gee, Sally Young.

## SUNFISH Working Group

**Principal investigators (in bold) and site study personnel**

*Belgium*: **Aurore Daron M.D**.**,** Stéphanie Delstanche M.D., Erica Marucco Fuentes M.D.; Ophthalmologists: Romain Bruninx, Ariane Milet; Physiotherapists: Fabian Dal Farra, Olivier Schneider; **Nicolas Deconinck, M.D.,** Ophthamologists: Irina Balikova, M.D., Patricia Delbeke M.D., Inge Joniau M.D.; Physiotherapists: Valentine Tahon, Sylvia Wittevrongel; Study coordinator: Elke De Vos; **Nathalie Goemans, M.D.,** Ingele Casteels M.D., Liesbeth De Waele M.D.; Ophthalmologists: Irina Balikova, Catherine Cassiman, Lies Prove; Physiotherapists: Sophie Huyskens, David Kinoo, Lisa Vancampenhout, Marleen Van Den Hauwe, Annelies Van Impe; *Brazil*: **Alexandra Prufer de Queiroz Campos Araujo M.D.,** Aline Chacon Pereira M.D., Flávia Nardes M.D.; Ophthamologists: Lorena Haefeli, Julia Rossetto; Physiotherapists: Marcos Ferreira Rebel, Jaqueline Almeida Pereira; *Canada*: **Craig** **Campbell M.D.,** Ophthalmologists: Sapna Sharan; Physiotherapists: Wendy McDonald, Cheryl Scholtes; **Jean Mah M.D.,** Maria Sframeli M.D., Claudia Stancanelli M.D.; Ophthalmologists: William Astle; Physiotherapists: Angela Chiu, Vanessa D’Souza, Jane Hagel; **Maryam Oskoui M.D.,** Ophthalmologists: Raquel Beneish, Gaela Cariou-Palmer, Connie Pham, Daniela Toffoli; Physiotherapists: Stephanie Arpin, Sarah Turgeon Desilets; *China*: **Yi Wang M.D.,** Chaoping Hu M.D., Jianfeng Huang M.D., Shuizhen Zhou M.D.; Ophthalmologists: Chen Qian, Li Shen, Ying Xiao, Zhenxuan Zhou; Physiotherapists: Hui Li, Sujuan Wang; **Hui Xiong M.D.,** Xingzhi Chang M.D., Hui Dong M.D., Ying Liu M.D., Tian Sang M.D., Cuijie Wei M.D., Jing Wen M.D.; Ophthalmologists: Yiwen Cao, Xingyao Ly, Jing Wen, Jingjing Zhao; Physiotherapists: Wenzhu Li, Lun Qin; *Croatia*: **Nina Barisic M.D.,** Ophthalmologists: Ivan Celovec, Martina Galiot Delic, Petra Kristina Ivkić, Nenad Vukojević; Physiotherapists: Ivana Kern, Boris Najdanovic, Marin Skugor; *France*: **Laurent Servais, M.D., Odile Boespflug-Tanguy M.D.,** Elena Gargaun M.D., Teresa Gidaro M.D., Andreea Seferian M.D; Ophthalmologists: Brigitte Audebert, Emmanuel Barreau, Elodie Da Cunha, Céline Lambotin, Nabila Mnafek, Marta Milkova Momtchilova, Helene Peche, Beatrice Pelosse, Flavie Rocher, Carole Valherie; Physiotherapists: Stephanie Gilabert, Allison Grange, Charlotte Lilien, Darko Milascevic, Ariadna Perticari, Shotaro Tachibana; **Jean Marie Cuisset M.D.,** **Jean-Baptiste Davion M.D.,** Stephanie Coopman M.D.**;** Ophthalmologists: Ikram Bouacha, Philippe Debruyne, Sabine Defoort, Gilles Derlyn, Florian Leroy; Physiotherapists: Loïc Danjoux, Claire Dedobbeleer-Brebion, Julie Guilbaud, Pauline Poreye; **Isabelle** **Desguerre M.D.,** Christine Barnérias M.D., Michaela Semeraro M.D.; Ophthalmologists: Dominique Bremond-Gignac, Lenaic Bruere, Maxence Rateaux, Matthieu Robert; Physiotherapists: Elodie Deladrière, Virginie Germa; **Yann Pereon M.D.,** Armelle Magot M.D., Sandra Mercier M.D.; Ophthalmologists: Fanny Billaud, Chloe Couret, Rudy Konig, Guylene Le Meur; Physiotherapists: Camille Hochet, Lucie Le Goff, Guy Letellier M.D.; **Carole** **Vuillerot M.D.,** Ophthalmologists: Nabil Bouzid, Aude Taleb; Physiotherapists: Aurélie Barriere, Marie Tinat; *Germany*: **Janbernd Kirschner M.D., Sabine Borell M.D.,** Ophthalmologists: Gabriela Arndt, Jan Bollig, Michelle Dreesbach, Lutz Joachimsen, Wolf Lagréze, Bettina Michaelis, Fanni Molnar, Dorina Seger, Julia Stifter; Physiotherapists: Juliane Hug, Sibylle Vogt; *Italy*: **Enrico** **Bertini M.D.,** Adele D’Amico M.D., Susana Livadiotti M.D., Guiseppe Pontrelli M.D., Alessandra Simonetti M.D.; Ophthalmologists: Rosa Parilla, Sergio Petroni, Paola Valente; Physiotherapists: Anna Maria Bonetti, Adelina Carlesi, Giulia Colia, Irene Mizzoni; **Claudio Bruno M.D.,** Ophthalmologists: Enrico Priolo, Giuseppe Rao, Enrica Spaletra, Lorenza Sposetti, Elisa Tassara; Physiotherapists: Valentina Lanzillotta, Simone Morando, Paola Tacchetti, Ambra Zuffi; **Giacomo Pietro Comi M.D.,** Roberta Brusa M.D., Stefania Corti M.D., Velardo Daniele M.D., Alessandra Govoni M.D., Francesca Magri M.D., Eleonora Mauri M.D.; Ophthalmologists: Valeria Minorini, Silvia Gabriella Osnaghi; Physiotherapists: Francesca Abbati, Federica Fassini, Michaela Foa, Amalia Lopopolo, Elisa Minuti; **Giovanni Baranello, M.D., Riccardo Masson M.D.,** Emanuela Pagliano M.D., Maria Barbara Pasanisi M.D.; Ophthalmologists: Stefania Bianchi Marzoli, Diletta Santarsiero, Myriam Garcia Sierra, Gemma Tremolada; Physiotherapists: Maria Teresa Arnoldi, Marta Vigano, Riccardo Zanin; **Eugenio Mercuri M.D.,** Ophthalmologists: Giulia Maria Amorelli, Costanza Barresi, Gugliemo D’Amico, Lorenzo Orazi; Physiotherapists: Giorgia Coratti, Roberto De Sanctis; *Japan*: **Kazuhiro Haginoya M.D.,** Ophthalmologists: Noriko Himori, Takayuki Takeshita, Sayaka Yoshida; Physiotherapists: Atsuko Kato, Yuko Morishita; **Ryutaro Kira M.D.,** Ophthalmologists: Kiyomu Akiyama, Miwako Goto, Yujiro Mori, Misato Okamoto, Saki Tsutsui; Physiotherapists: Yuta Takatsuji, Aya Tanaka; **Hirofumi Komaki M.D.,** Ophthalmologists: Miina Omori; Physiotherapists: Ippei Suzuki, Mizuki Takeuchi, Daisuke Todoroki, Hiroyuki Yajima; **Seji Watanabe M.D., Tomoko Matsubayashi M.D.,** Opthamologists: Atsuko Matsuhisa; Physiotherapists: Emi Inakazu, Hiroe Nagura, Akira Suzuki; **Hitoshi Osaka M.D.,** Ophthalmologists: Meri Watanabe; Physiotherapists: Emi Takahashi, Keita Takaishi, Manami Usui; **Nobutsune Ishikawa M.D.,** Ophthalmologists: Yousuke Harada; Physiotherapists: Kenishi Fudeyasu, Kazuhiko Hirata, Kana Michiue, Kazuyuki Ueda; **Kayoko Saito M.D.,** **Reiko Arakawa M.D.,** Ophthalmologists: Shigeko Yashiro; Physiotherapists: Hiroko Kanno, Hidemi Kono, Yukiko Nishigaki, Yuji Sato, Maiko Seki; **Nozomi Sano M.D.,** Ophthalmologists: Akinori Uemura; Physiotherapists: Koji Fukuyama, Yuki Matsumoto, Hirofumi Miyazaki; **Minoru Shibata M.D.,** Ophthalmologists: Shohei Eda, Tomoko Terai; Physiotherapists: Kyoko Kobayashi, Yukie Nakamura; **Yasuhiro Takeshima M.D.,** Ophthalmologist: Fumi Gomi, Yuka Hosotani, Mana Okamoto; Physiotherapist: Moe Kuma, Ai Yanagida; *Poland*: **Anna** **Kostera-Pruszczyk M.D.,** Anna Fraczek M.D., Maria Jedrzejowska M.D., Anna Lusakowska M.D.; Ophthalmologists: Agnieszka Czeszyk-Piotrowicz, Wojciech Hautz, Klaudia Rakusiewicz, Andrzej Zawada; Physiotherapists: Malgorzata Burlewicz, Zuzanna Gierlak-Wojcicka, Malwina Kępa, Adam Sikorski, Marcin Sobieraj, Jarosław Steinhagen; **Maria** **Mazurkiewicz-Bełdzińska M.D.,** Anna Lemska M.D., Agnieszka Matheisel M.D., Sandra Modrzejewska M.D., Marta Zawadzka M.D.; Ophthalmologists: Mateusz Koberda, Natalia Kubiak, Urszula Stodolska-Koberda, Agnieszka Waśkowska; Physiotherapists: Jagoda Kolendo, Agnieszka Sobierajska-Rek; **Barbara Steinborn M.D.,** Jędrzej Fliciński M.D., Agnieszka Wencel-Warot M.D., Anna Winczewska-Wiktor M.D.; Ophthalmologists: Magdalena Dalz, Julia Grabowska, Wojciech Hajduk, Jacek Idziak, Justyna Janasiewicz-Karachitos, Monika Klimas, Marcin Stopa; Physiotherapists: Ewa Gajewska, Beata Pusz; *Russia*: **Dmitry Vlodavets M.D.,** Evgenia Melnik M.D.; Ophthalmologists: Natalya Leppenen, Nataliya Yupatova; Physiotherapists: Elena Litvinova, Anastasya Monakhova, Yulia Papina, Olga Shidlovsckaia; *Serbia*: **Vedrana** **Milic Rasic M.D.,** Vesna Brankovic M.D., Ana Kosac M.D., Ophthalmologists: Olivera Djokic, Vesna Jakšić, Ana Pepic; Physiotherapists: Jelena Martinovic; *Spain*: **Francina Munell Casadesus M.D.,** Eduardo Tizzano M.D.; Ophthalmologists: Nieves Martín Begué, Charlotte Wolley Dod, Silvia Alarcón Portabella, Olaia Subira; Physiotherapists: Bernat Planas Pascual, Esther Toro Tamargo; **Marcos Madruga Garrido M.D.,** Mercedes Lopez Lobato M.D, Mirella Gaboli M.D.; Ophthalmologists: Begoña Balboa Huguet, Eva María Parra Oviedo; Physiotherapists: José David Medina Romero, Marta Peña Salinas; **Andrés Nascimento Osorio M.D.,** Ophthalmologists: Ana Díaz Cortés, Enrique Jiménez Gañan, Joan Prat, Simone Dowon Suh; Physiotherapists: Julita Medina, Obdulia Moya, Nuria Padros, Sandra Roca Urraca; **Samuel Pascual Pascual M.D.,** Ophthalmologists: Sofía de Manuel, Susana Noval Martin; Physiotherapists: Paul Burnham, Sandra Espinosa Garcia, Mercedes Martinez Moreno; *Turkey*: **Haluk Topaloglu, M.D., Ibrahim Oncel M.D.,** Nesibe Eroglu Ertugrul M.D., Mina Gharibzadeh Hizel M.D., Bahadir Konuskan M.D., Selen Serel Arslan M.D., Elmas Ebru Yalcin M.D.; Ophthalmologists: Bora Eldem, Sibel Kadayıfçılar; Physiotherapists: Ipek Alemdaroglu, Aynur Ayse Karaduman, Oznur Tunca Yilmaz; *USA*: **Claudia Chiriboga M.D.,** Ophthalmologist: Ma Edylin M. Bautista, Steven Kane; Physiotherapists: John Lee, Donnielle Rome-Martin, Rachel Salazar; **John W. Day M.D.,** Ophthalmologists: Shannon Beres, Lisa Greer; Physiotherapists: Tina Duong, Richard Gee, Chelsea Macpherson, Sally Young.

## JEWELFISH Working Group

**Principal investigators (in bold) and site study personnel**

*Belgium*: **Nicolas Deconinck M.D.,** Opthalmologists: Irina Balikova M.D., Inge Joniau M.D.; Physiotherapists: Valentine Tahon, Sylvia Wittevrongel; **Nathalie Goemans M.D.,** Ophthalmologists: Irina Balikova, Catherine Cassiman, Lies Prove; Physiotherapists: Sophie Huyskens, David Kinoo, Lisa Vancampenhout, Marleen Van Den Hauwe, Annelies Van Impe; *France*: **Claude Cances M.D.,** Ophthalmologists: Safa El Hout, Félix Fremont, Clement Gomene, Pauline Meyer, Jasmine Pechmeja, Anthony Seigner; Physiotherapists: Valérie Bellio; Olaia Gil; **Jean Marie Cuisset M.D., Jean-Baptiste Davion M.D.,** Ophthalmologists: Ikram Bouacha, Philippe Debruyne, Gilles Derlyn, Sabine Defoort, Florian Leroy; Physiotherapists: Loïc Danjoux, Claire Dedobbeleer-Brebion, Julie Guilbaud, Pauline Poreye; **Isabelle Desguerre M.D.,** Ophthalmologists: Dominique Bremond-Gignac, Lenaic Bruere, Maxence Rateaux, Matthieu Robert; Physiotherapists: Elodie Deladrière, Virginie Germa; **Carole Vuillerot M.D.,** Ophthalmologists: Charlotte Es-Saidi, Bénédicte Sibille-Dabadi, Quentin Veillerot; Physiotherapists: Aurélie Barriere, Marie Tinat; **Ulrike** **Walther-Louvier M.D.,** Ophthalmologists: Dr Pierre-André Duval, Pascale Caradec, Isabelle Cavailhes; Physiotherapists: Florina Dragan, Fanette Pelatan, Blandine Puyhaubert, Souad Touati; *Germany*: **Janbernd Kirschner M.D., Sabine** **Borell M.D.,** Ophthalmologists: Gabriela Arndt, Jan Bollig, Nikolai Gross, Lutz Joachimsen, Wolf Lagréze, Bettina Michaelis, Fanni Molnar, Dorina Seger, Julia Stifter; Physiotherapists: Juliane Hug, Sibylle Vogt; **Ulrike Schara M.D., Heike** **Kölbel M.D., Tim Hagenacker M.D.,** Ophthalmologists: Anja Eckstein, Dirk Dekowski, Juliane Doehr, Ulrike Kaiser, Heike Mayer, Michael Oeverhaus, Mareile Stoehr; Physiotherapists: Barbara Andres, Uta Leyener; *Italy*: **Enrico Bertini M.D.,** Adele D’Amico M.D., Ophthalmologists: Rosa Parilla, Sergio Petroni, Paola Valente; Physiotherapists: Anna Maria Bonetti, Adelina Carlesi, Giulia Colia, Irene Mizzoni; **Claudio Bruno M.D.,** Giorgia Brigati M.D.; Ophthalmologists: Enrico Priolo, Giuseppe Rao, Enrica Spaletra, Lorenza Sposetti, Elisa Tassara; Physiotherapists: Valentina Lanzillotta, Simone Morando, Paola Tacchetti, Ambra Zuffi; **Giacomo Pietro Comi M.D.,** Ophthalmologists: Silvia Gabriella Osnaghi, Valeria Minorini; Physiotherapists: Francesca Abbati, Federica Fassini, Michaela Foa, Amalia Lopopolo, Elisa Minuti; **Eugenio Mercuri M.D.,** Ophthalmologists: Giulia Maria Amorelli, Costanza Barresi, Gugliemo D’Amico, Lorenzo Orazi; Physiotherapists: Giorgia Coratti, Roberto De Sanctis; **Giuseppe Vita M.D.,** Maria Sframeli M.D., Gian Luca Vita M.D.; Ophthalmologists: Pasquale Aragona, Leandro Inferrera, Daniela Montanini, Elisa Imelde Postorino; Physiotherapists: Vincenzo Di Bella, Concetta Donato; *Netherlands*: **Ludo Van der Pol M.D.,** Ophthalmologists: Jos Aalbers, Joke de Boer, Pascale Cooijmans, Saskia Imhof; Physiotherapists: Bart Bartels, Thijs Ruyten, Danny Van Der Woude; *Poland*: **Anna Kostera-Pruszczyk M.D.,** Ophthalmologists: Katarzyna Kierzkowska, Beata Klimaszewska, Dominika Romańczak, Justyna Szybka, Bartosz Wolinski, Anna Wolska; Physiotherapists: Malgorzata Burlewicz, Zuzanna Gierlak-Wojcicka, Malwina Kępa, Adam Sikorski, Marcin Sobieraj, Jarosław Steinhagen; *Swizterland*: **Dirk Fischer M.D.,** Patricia Hafner M.D., Bettina Henzi M.D., Andrea Klein M.D., Sara Nagy M.D., Simone Schmidt M.D.; Opthalmologists: Konstantin Gugleta, Akos Kusnyerik, Patricia Siems; Physiotherapists: Sabina Akos, Sabrina Ernst, Nora Frei, Verena Kreiliger, Christine Seppi, Christine Wondrusch Haschke; *United Kingdom*; **Volker Straub M.D.,** **Michela Guglieri M.D.,** Ophthalmologists; Richard Bell, Liam Grant, Gemma Loudon, Joseph McLachlan, Mahmoud Nassar, Stuart Page, Michael Patrick Clarke, Aedheen Regan, Karl Southerton, Sam Sparrowhawk; Physiotherapists: Anna Mayhew, Dionne Moat, Robert Muni Lofra; **Deepak Parasuraman M.D.,** Ophthalmologists: Suaad Alasow, Simone Bruschi, Andrew Castle, Laura Croxton, Abdul-Jabbar Ghauri, Saima Naqvi, Nicola Patt; Physiotherapists: Heather Mcmurchie, Rosanna Rabb; **Maria Scoto M.D.,** Federica Trucco M.D.; Ophthalmologists: Robert H Henderson M.D., Roopen Kukadia, Will Moore, Nakita Tanwar; Physiotherapists: Mario Iodice, Evelin Milev, Efthymia Panagiotopoulou, Catherine Rye, Victoria Selby, Amy Wolfe; *United States of America*: **Claudia Chiriboga M.D.,** Ophthalmologists: Ma Edylin M. Bautista, Eileen Frommer, Steven Kane, Noelle Pensec; Physiotherapists: John Lee, Donnielle Rome-Martin, Rachel Salazar; **Basil T. Darras M.D.,** Ophthalmologists: Lucia Ambrosio, Anna Maria Baglieri, Anne Fulton; Physiotherapists; Courtney Dias, Elizabeth Maczek, Elizabeth Mirek; Amy Pasternak; **John W. Day M.D.,** Ophthalmologists: Shannon Beres, Lisa Greer; Physiotherapists: Tina Duong, Richard Gee, Chelsea Macpherson, Sally Young; **Richard Finkel M.D., Aledie Navas Nazario M.D.,** Ophthalmologists: Leonardo Aldarondo, Airaj Fasiuddin, Ana Rivera; Physiotherapists: Matthew Civitello, Julie Wells.

# S1B. FIREFISH ELIGIBILITY CRITERIA

## Inclusion criteria

Infants met the following criteria for study entry –

1. Males and females aged between 28 days (1 month) and 210 days (7 months) (inclusive) at enrollment. For the first three patients enrolled in Part 1, age was between 150 days (5 months) and 210 days (7 months) inclusive and a minimum body weight of 7 kg was required for the first patient only. Enrollment was defined as the moment when a patient had their dosing number assigned.
2. A legally authorized representative who was able to consent for the patient according to the International Council for Harmonisation of Technical Requirements for Pharmaceuticals for Human Use (ICH) and local regulations.
3. Gestational age of 37 to 42 weeks.
4. Confirmed diagnosis of 5q-autosomal recessive spinal muscular atrophy (SMA), including:
   1. genetic confirmation of homozygous deletion or compound heterozygosity predictive of loss of function of the survival of motor neuron 1 (*SMN1*) gene
   2. clinical history, signs or symptoms attributable to Type 1 SMA, i.e., hypotonia, absent deep tendon reflex and/or tongue fasciculations with onset after the age of 28 days, but prior to the age of 3 months (inclusive), and inability to sit independently (without support) at the time of screening.
5. Patient had two *SMN2* gene copies, as confirmed by central testing.
6. Body weight ≥3rd percentile for age, using appropriate country-specific guidelines (for the first patient only: >7 kg).
7. Receiving adequate nutrition and hydration (with or without gastrostomy) at the time of screening, in the opinion of the investigator.
8. Adequately recovered from any acute illness at the time of screening and considered well enough to participate in the opinion of the investigator.
9. Medical care met, in the opinion of the investigator, locally accepted standard of care.
10. Able to safely travel to the study site for the whole duration of the study and according to the frequency of required study visits, in the opinion of the investigator. Air travel was strongly discouraged. The overall condition and situation (including geographical) of the patient was evaluated and the decision taken by the investigator prior to enrollment.
11. Had a stable home situation with a consistent caregiver.
12. Would be able to complete all study procedures, measurements and visits, and the parent or caregiver of the patient, in the opinion of the investigator, had adequately supportive psychosocial circumstances.
13. If not already in place at the time of screening, parent or caregiver of patient was willing to consider nasogastric, naso-jejunal or gastrostomy tube placement during the study to maintain safe hydration, nutrition and treatment delivery, as recommended by the investigator.
14. If not already in place at the time of screening, parent or caregiver of infant was willing to consider the use of non-invasive ventilation during the study, as recommended by the investigator.

## Exclusion criteria

Patients who met any of the following criteria were excluded from study entry –

1. Inability to meet study requirements.
2. Concomitant or previous participation in any investigational drug or device study within 90 days prior to screening or 5 half-lives, whichever was longer.
3. Concomitant or previous administration of an *SMN2*-targeting antisense oligonucleotide, *SMN2* splicing modifier or gene therapy either in a clinical study or as part of medical care.
4. Any history of cell therapy.
5. Hospitalization for a pulmonary event within the last 2 months or planned at the time of screening.
6. Unstable gastrointestinal, renal, hepatic, endocrine or cardiovascular system diseases.
7. In the opinion of the investigator, inadequate venous or capillary blood access for the study procedures.
8. Required invasive ventilation or tracheostomy.
9. Required awake non-invasive ventilation or with awake hypoxemia (SaO_2_ < 95%) with or without ventilator support.
10. A history of respiratory failure or severe pneumonia and had not fully recovered their pulmonary function at the time of screening.
11. Multiple or fixed contractures and/or hip subluxation or dislocation at birth.
12. Presence of non-SMA-related concurrent syndromes or diseases.
13. Confirmed (two consecutive measurements) systolic blood pressure or diastolic blood pressure outside the 95th percentile for age; resting heart rate <70 bpm or >170 bpm.
14. Presence of clinically relevant electrocardiogram (ECG) abnormalities before study drug administration; corrected QT interval using Bazett’s method (QTcB) >460 ms; personal or family history (first-degree relatives) of congenital long QT syndrome indicating a safety risk for patients as determined by the investigator. First-degree atrioventricular block or isolated right bundle branch block are allowed.
15. History of malignancy if not considered cured.
16. Any major illness within 1 month before the screening examination or any febrile illness within 1 week prior to screening and up to first dose administration.
17. Taking any nutrients known to modulate cytochrome 3A (CYP3A) activity (e.g., grapefruit juice; Seville orange) within 2 weeks prior to administration of study drugs.
18. The infant (and the mother, if breastfeeding the infant):
    1. any inhibitor of CYP3A4 taken within 2 weeks (or within five times the elimination half-life, whichever is longer) prior to dosing, including but not limited to: ketoconazole, miconazole, itraconazole, fluconazole, erythromycin, clarithromycin, ranitidine, cimetidine
    2. any inducer of CYP3A4 taken within 4 weeks (or within five times the elimination half-life, whichever is longer) prior to dosing, including but not limited to: rifampicin, rifabutin, glucocorticoids, carbamazepine, phenytoin, phenobarbital, St. John's wort
    3. any organic cation transporter-2 (OCT-2) and multidrug and toxin extrusion (MATE) substrates were avoided (including but not limited to: amantadine, cimetidine, memantine, amiloride, famotidine, metformin, pindolol, ranitidine, procainamide, varenicline, acyclovir, ganciclovir, oxaliplatin, cephalexin, cephradine, fexofenadine)
    4. any known flavin monooxygenase 1 (FMO1) or FMO3 inhibitors or substrates.
19. Clinically significant abnormalities in laboratory test results e.g., Grade >1 anemia, alanine aminotransferase (ALT) values exceeding 1.5 x the upper limit of normal unless the elevated ALT level was considered of muscular origin (i.e., in the absence of other evidence of liver disease which was confirmed by elevated creatine kinase and lactate dehydrogenase). Out of range creatine kinase levels were reviewed in light of the underlying SMA pathology of the patient; elevated levels per se did not disqualify the patient from the study. In the case of uncertain or questionable results, tests performed during screening could be repeated before enrollment to confirm eligibility.
20. Ascertained or presumptive hypersensitivity (e.g., anaphylactic reaction) to risdiplam or to the constituents of its formulation.
21. Concomitant disease or condition that could have interfered with, or treatment of which might have interfered with, the conduct of the study, or that would have, in the opinion of the investigator, posed an unacceptable risk to the patient in this study.
22. Therapeutic use, defined as use for ≥8 weeks, of the following medications within 90 days prior to enrollment: riluzole, valproic acid, hydroxyurea, sodium phenylbutyrate, butyrate derivatives, creatine, carnitine, growth hormone, anabolic steroids, probenecid, agents anticipated to increase or decrease muscle strength, agents with known or presumed histone deacetylase inhibitory effect, medications known to or suspected of causing retinal toxicity (e.g., deferoxamine, topiramate, latanoprost, niacin, rosiglitazone, tamoxifen, canthaxanthin, sildenafil, and interferon) and medications with known phototoxicity liabilities (e.g., oral retinoids including over-the-counter formulations, amiodarone, phenothiazines and use of minocycline). Shorter use of any of these drugs within 90 days prior to enrollment was reviewed on a case-by-case basis and discussed between the sponsor and the investigator, who jointly made the decision on whether the patient could be enrolled in the study (infants who were on inhaled corticosteroids, administered either through a nebulizer or an inhaler, were allowed in the study).
    1. Infants were not to begin treatment with the above medications after initiating study drug.
23. Recently initiated treatment (within <6 weeks prior to enrollment) with oral salbutamol or another β2-adrenergic agonist taken orally was not allowed. Infants who had been on oral salbutamol (or another β2-adrenergic agonist) for ≥6 weeks before screening and had shown good tolerance, were allowed. The dose of β2-adrenergic agonist was to remain stable as much as possible for the duration of the study. Use of inhaled β2-adrenergic agonists (e.g., for the treatment of asthma) was allowed.
24. Prior use (at any time in the patients’ lives) and/or anticipated need for quinolines (chloroquine and hydroxychloroquine), thioridazine, vigabatrin, retigabine, or any other drug known to cause retinal toxicity during the study did not allow participation in the trial. Infants exposed to chloroquine, hydroxycholoroquine, thioridazine, vigabatrin, retigabine or drugs with known retinal toxicity given to mothers during pregnancy (and lactation) were not to be enrolled.
25. Recent history (<6 months) of ophthalmologic diseases (e.g., glaucoma not controlled by treatment, central serous retinopathy, inflammatory/infectious retinitis unless clearly inactive, retinal detachment, intraocular trauma, retinal dystrophy or degeneration, optic neuropathy, or optic neuritis) that would have interfered with the conduct of the study as assessed by an ophthalmologist. Any other abnormalities detected with optical coherence tomography (OCT) at screening (e.g., retinal layer abnormalities, edema, cystic or atrophic changes) were to be discussed with the investigator, ophthalmologist, and with the sponsor, who jointly made the decision on whether the infant could be enrolled in the study. Infants in whom OCT measurement of sufficient quality could not be obtained at screening were not enrolled.

# S1C. SUNFISH ELIGIBILITY CRITERIA

## Inclusion criteria

Patients must meet the following criteria for study entry –

1. Males and females 2–25 years of age inclusive (at screening).
2. For Part 1: Type 2 or 3 SMA ambulant or non-ambulant. For Part 2: Type 2 or 3 SMA non-ambulant. Non-ambulant is defined as not having the ability to walk unassisted (i.e., without braces, assisted devices such as canes, crutches or calipers, or person/hand-held assistance) for 10 minutes or more.
3. Confirmed diagnosis of 5q-autosomal recessive SMA, including:
   1. genetic confirmation of homozygous deletion or heterozygosity predictive of loss of function of the *SMN1* gene
   2. clinical symptoms attributable to Type 2 or Type 3 SMA.
4. For non-ambulant patients in Part 2:
   1. Revised Upper Limb Module (RULM) Entry Item A (Brooke score) ³ 2 (i.e., “Can raise 1 or 2 hands to the mouth, but cannot raise a 200 g weight in it to the mouth”)
   2. ability to sit independently (i.e., scores ≥1 on Item 9 of the 32-item Motor Function Measure “with support of one or both upper limbs maintains the seated position for 5 seconds”).
5. Able and willing to provide written informed consent and to comply with the study protocol according to ICH and local regulations. Alternatively, a legally authorized representative must be able to consent for the patient according to ICH and local regulations and assent must be given whenever possible.
6. Negative blood pregnancy test at screening (all women of childbearing potential, including those who have had a tubal ligation), and agreement to comply with measures to prevent pregnancy and restrictions on sperm donation, as below:
   1. for women who are not prematurely menopausal (≥12 months of non-therapy-induced amenorrhea) or surgically sterile (absence of ovaries and/or uterus): agreement to remain abstinent (refrain from heterosexual intercourse) or to use two adequate methods of contraception, including at least one method with a failure rate of <1% per year, during the treatment period and for at least 28 days after the last dose of study drug. The reliability of sexual abstinence needs to be evaluated in relation to the duration of the clinical trial and the preferred and usual lifestyle of the patient. Periodic abstinence (e.g., calendar, ovulation, symptothermal, or post-ovulation methods) and withdrawal are not acceptable methods of contraception. Barrier methods must always be supplemented with the use of a spermicide
   2. examples of contraceptive methods with a failure rate of <1% per year include bilateral tubal ligation, male sterilization, established and proper use of hormonal contraceptives that inhibit ovulation, hormone-releasing intrauterine devices, and copper intrauterine devices
   3. for men: agreement to remain abstinent (refrain from heterosexual intercourse) or use contraceptive measures and agreement to refrain from donating sperm, as defined below:
      1. with female partners of childbearing potential, men must remain abstinent or use a condom plus an additional contraceptive method that together result in a failure rate of <1% per year during the treatment period and for at least 4 months after the last dose of study drug. Men must refrain from donating sperm during this same period. This period is required for small molecules with potential for genotoxic effect and includes spermatogenic cycle duration and drug elimination process
      2. with pregnant female partners, men must remain abstinent or use a condom during the treatment period and for at least 28 days after the last dose of study drug. The reliability of sexual abstinence needs to be evaluated in relation to the duration of the clinical trial and the preferred and usual lifestyle of the patient. Periodic abstinence (e.g., calendar, ovulation, symptothermal, or post-ovulation methods) and withdrawal are not acceptable methods of contraception.

## Exclusion criteria

Patients who meet any of the following criteria will be excluded from study entry –

1. Inability to meet study requirements.
2. Concomitant or previous participation in any investigational drug or device study within 90 days prior to screening, or five half-lives of the drug, whichever is longer.
3. Concomitant or previous administration of an *SMN2*-targeting antisense oligonucleotide, *SMN2* splicing modifier or gene therapy either in a clinical study or as part of medical care.
4. Any history of cell therapy.
5. Hospitalization for a pulmonary event within the last 2 months or planned at time of screening.
6. Surgery for scoliosis or hip fixation in the 1 year preceding screening or planned within the next 18 months.
7. Unstable gastrointestinal, renal, hepatic, endocrine, or cardiovascular system diseases as considered to be clinically significant by the investigator.
8. Lactating women.
9. Suspicion of regular consumption of drug of abuse.
10. Positive urine test for drugs of abuse or alcohol at screening or baseline visit (adolescents and adults only).
11. Cardiovascular, blood pressure and heart rate:
    1. adults: sustained resting systolic blood pressure (SBP) >140 mmHg or <80 mmHg, and/or diastolic blood pressure (DBP) >90 mmHg or <40 mmHg; a resting heart rate <45 bpm or >100 bpm
    2. adolescents (12−17 years of age): SBP and/or DBP outside the 95th percentile for age; resting heart rate <50 bpm or >100 bpm
    3. children (6−11 years of age): SBP and/or DBP outside the 95th percentile for age; resting heart rate <60 bpm or >120 bpm
    4. children (2−5 years of age): SBP and/or DBP outside the 95th percentile for age; resting heart rate <70 bpm or >140 bpm.
12. Presence of clinically significant ECG abnormalities before study drug administration (e.g., second or third degree AV block, confirmed QTcF >460 ms for patients age >10 years or QTcB >460 ms for children up to age 10 years as Bazett’s correction is more appropriate in young children) from average of triplicate measurement or cardiovascular disease (e.g., cardiac insufficiency, coronary artery disease, cardiomyopathy, congestive heart failure, family history of congenital long QT syndrome, family history of sudden death) indicating a safety risk for patients as determined by the investigator.
13. History of malignancy if not considered cured.
14. Significant risk for suicidal behavior, in the opinion of the investigator as assessed by the Columbia Suicide Severity Rating Scale (>6 years of age).
15. Any major illness within 1 month before the screening examination or any febrile illness within 1 week prior to screening and up to first dose administration.
16. Any organic cation transporter 2 and multidrug and toxin extrusion substrates within 2 weeks before dosing (including but not limited to: amantadine, cimetidine, memantine, amiloride, famotidine, metformin, pindolol, ranitidine, procainamide, varenicline, acyclovir, ganciclovir, oxaliplatin, cephalexin, cephradine, fexofenadine).
17. Use of the following medications within 90 days prior to randomization: riluzole, valproic acid, hydroxyurea, sodium phenylbutyrate, butyrate derivatives, creatine, carnitine, growth hormone, anabolic steroids, probenecid, agents anticipated to increase or decrease muscle strength, agents with known or presumed histone deacetylase (HDAC) inhibitory effect, and medications with known phototoxicity liabilities (e.g., oral retinoids including over the counter formulations, amiodarone, phenothiazines and chronic use of minocycline). (Patients who are on inhaled corticosteroids, administered either through a nebulizer or an inhaler, will be allowed in the study).
18. Recently initiated treatment (within <6 months prior to randomization) with oral salbutamol or another β2-adrenergic agonist taken orally is not allowed. Patients who have been on oral salbutamol (or another β2-adrenergic agonist) for ≥6 months before randomization and have shown good tolerance are allowed. The dose of β2-adrenergic agonist should remain stable as much as possible for the duration of the study. Use of inhaled β2-adrenergic agonists (e.g., for the treatment of asthma) is allowed.
19. Any prior use of chloroquine, hydroxychloroquine, retigabine, vigabatrin or thioridazine, is not allowed. Use of other medications known to or suspected of causing retinal toxicity within 1 year prior to randomization is not allowed.
20. Clinically significant abnormalities in laboratory test results, e.g., ALT values exceeding 1.5-fold the upper limit of normal, unless the elevated ALT level is considered of muscular origin (i.e., in the absence of other evidence of liver disease) which is supported by elevated creatine kinase and lactate dehydrogenase (LDH). Out of range creatine kinase levels should be reviewed in light of the underlying SMA pathology of the patient; elevated levels per se do not disqualify the patient from the study. In the case of uncertain or questionable results, tests performed during screening may be repeated before randomization to confirm eligibility.
21. Donation or loss of blood ≥10% of blood volume within 3 months prior to screening.
22. Ascertained or presumptive hypersensitivity (e.g., anaphylactic reaction) to RO7034067 or to the constituents of its formulation (RO7034067 Investigator’s Brochure).
23. Concomitant disease or condition that could interfere with, or treatment of which might interfere with, the conduct of the study, or that would, in the opinion of the investigator, pose an unacceptable risk to the patient in this study.
24. Recent history (less than 1 year) of ophthalmologic diseases (e.g., glaucoma not controlled by treatment, central serous retinopathy, inflammatory/infectious retinitis unless clearly inactive, retinal detachment, retinal surgery, intraocular trauma, retinal dystrophy or degeneration, optic neuropathy, or optic neuritis) that would interfere with the conduct of the study as assessed by an ophthalmologist. Any other abnormalities detected at screening (e.g., retinal layer abnormalities, edema, cystic or atrophic changes) must be discussed with the investigator, ophthalmologist, and with the sponsor, who will jointly make the decision if the patient may be enrolled in the study. Patients in whom OCT measurement of sufficient quality cannot be obtained at screening will not be enrolled.
25. Patients requiring invasive ventilation or tracheostomy.

# S1D. JEWELFISH ELIGIBILITY CRITERIA

## Inclusion criteria

Patients must meet the following criteria for study entry –

1. Males and females 6 months–60 years of age inclusive (at screening).
2. Confirmed diagnosis of 5q-autosomal recessive SMA, including:
   1. genetic confirmation of homozygous deletion or heterozygosity predictive of loss of function of the *SMN1* gene
   2. clinical history, signs, or symptoms attributable to SMA.
3. Previous enrollment in Study BP29420 (MOONFISH) with the splicing modifier RO6885247 or previous treatment with any of the following:
   1. nusinersen (defined as having received ≥4 doses of nusinersen, provided that the last dose was received ≥90 days prior to screening)
   2. olesoxime (provided that the last dose was received ≤18 months and ≥90 days prior to screening)
   3. AVXS-101 (provided that the time of treatment was ≥12 months prior to screening).
4. Able and willing to provide written informed consent and to comply with the study protocol according to ICH and local regulations. Alternatively, a legally authorized representative must be able to give consent for the patient according to ICH and local regulations and assent must be given whenever possible.
5. Adequately recovered from any acute illness at the time of screening and considered well enough to participate in the opinion of the investigator.
6. For women of childbearing potential: negative blood pregnancy test at screening, agreement to remain abstinent (refrain from heterosexual intercourse) or use contraceptive measures, and agreement to refrain from donating eggs, as defined below:
   1. women must remain abstinent (refrain from heterosexual intercourse) or use two adequate methods of contraception, including at least one method with a failure rate of <1% per year, during the treatment period and for at least 28 days after the final dose of study drug. Women must refrain from donating eggs during this same period
   2. a woman is considered to be of childbearing potential if she is post-menarcheal, has not reached a post-menopausal state (≥12 continuous months of amenorrhea with no identified cause other than menopause), and has not undergone surgical sterilization (removal of ovaries and/or uterus). The definition of childbearing potential may be adapted for alignment with local guidelines or regulations
   3. examples of contraceptive methods with a failure rate of <1% per year include bilateral tubal ligation, male sterilization, established and proper use of hormonal contraceptives that inhibit ovulation, hormone-releasing intrauterine devices, and copper intrauterine devices
      1. a vasectomy is a highly effective birth control method provided that the partner is the sole sexual partner of the woman of childbearing potential trial participant and provided the vasectomized partner has received medical assessment of the surgical success
   4. the reliability of sexual abstinence should be evaluated in relation to the duration of the clinical trial and the preferred and usual lifestyle of the patient. Periodic abstinence (e.g., calendar, ovulation, symptothermal, or post-ovulation methods) and withdrawal are not acceptable methods of contraception. If required per local guidelines or regulations, locally recognized acceptable methods of contraception and information about the reliability of abstinence will be described in the local Informed Consent Form
   5. for men: agreement to remain abstinent (refrain from heterosexual intercourse) or use contraceptive measures and agreement to refrain from donating sperm, as defined below:
      1. with a female partner of childbearing potential, men must remain abstinent or use a condom plus an additional contraceptive method that together result in a failure rate of <1% per year during the treatment period and for at least 4 months after the final dose of study drug. Men must refrain from donating sperm during this same period. This period is required for small molecules with potential for genotoxic effect and includes the spermatogenic cycle duration and drug elimination process
      2. with a pregnant female partner, men must remain abstinent or use a condom during the treatment period and for at least 28 days after the final dose of study drug
      3. the reliability of sexual abstinence should be evaluated in relation to the duration of the clinical trial and the preferred and usual lifestyle of the patient. Periodic abstinence (e.g., calendar, ovulation, symptothermal, or post-ovulation methods) and withdrawal are not acceptable methods of contraception.
7. For patients aged 2 years or younger at screening:
   1. receiving adequate nutrition and hydration (with or without gastrostomy) at the time of screening, in the opinion of the investigator
   2. medical care meets local accepted standard of care, in the opinion of the investigator
   3. would be able to complete all study procedures, measurements and visits, and the parent or caregiver of the patient has adequately supportive psychosocial circumstances, in the opinion of the investigator
   4. parent or caregiver of patient is willing to consider nasogastric, naso-jejunal or gastrostomy tube placement, as recommended by the investigator, during the study (if not already in place at the time of screening) to maintain safe hydration, nutrition and treatment delivery
   5. parent or caregiver of patient is willing to consider the use of non-invasive ventilation, as recommended by the investigator during the study (if not already in place at the time of screening).

## Exclusion criteria

Patients who meet any of the following criteria will be excluded from study entry –

1. Inability to meet study requirements.
2. Concomitant participation in any investigational drug or device study. With the exception of studies of olesoxime, AVXS-101, or nusinersen: previous participation in any investigational drug or device study within 90 days prior to screening, or five half-lives of the drug, whichever is longer.
3. Any history of gene or cell therapy, with the exception of AVXS-101.
4. Unstable gastrointestinal, renal, hepatic, endocrine, or cardiovascular system diseases as considered to be clinically significant by the investigator.
5. Inadequate venous or capillary blood access for the study procedures, in the opinion of the investigator.
6. For patients aged <2 years, hospitalization for a pulmonary event within 2 months prior to screening and pulmonary function not fully recovered at the time of screening.
7. Lactating women.
8. Suspicion of regular consumption of drugs of abuse.
9. For adults and adolescents only, i.e., aged >12 years, positive urine test for drugs of abuse or alcohol at screening or Day 1 visit.
10. Cardiovascular, blood pressure, and heart rate:
    1. adults: sustained resting systolic blood pressure (SBP) >140 mmHg or <80 mmHg, and/or diastolic blood pressure (DBP) >90 mmHg or <40 mmHg; a resting heart rate <45 bpm or >100 bpm if considered to be clinically significant by the investigator
    2. adolescents (12‒17 years of age): SBP and/or DBP outside the 95th percentile for age; resting heart rate <50 bpm or >100 bpm if considered to be clinically significant by the investigator
    3. children (6–11 years of age): SBP and/or DBP outside the 95th percentile for age; resting heart rate <60 bpm or >120 bpm, if considered to be clinically significant by the investigator
    4. children (2–5 years of age): SBP and/or DBP outside the 95th percentile for age; resting heart rate <70 bpm or >140 bpm if considered to be clinically significant by the investigator
    5. children (6 months to <2 years of age): SBP and/or DBP outside the 95th percentile for age; resting heart rate <70 bpm or >170 bpm, if considered to be clinically significant by the investigator.
11. Presence of clinically significant ECG abnormalities before study drug administration (e.g., second or third degree AV block, confirmed QTcF >460 msec for patients aged ≥10 years, or QTcB >460 ms for children up to age 10 years (Bazett’s correction is more appropriate in young children) from the average of triplicate measurements, or cardiovascular disease (e.g., cardiac insufficiency, coronary artery disease, cardiomyopathy, congestive heart failure, family history of congenital long QT syndrome, family history of sudden death) indicating a safety risk for the patient as determined by the investigator.
12. History of malignancy if not considered cured.
13. For patients aged >6 years, significant risk for suicidal behavior, in the opinion of the investigator as assessed by the Columbia-Suicide Severity Rating Scale (C-SSRS).
14. Any major illness within 1 month before the screening examination or any febrile illness within 1 week prior to screening and up to first dose administration.
15. Use of any OCT-2 and MATE substrates within 2 weeks before dosing (including but not limited to: amantadine, cimetidine, memantine, amiloride, famotidine, metformin, pindolol, ranitidine, procainamide, varenicline, acyclovir, ganciclovir, oxaliplatin, cephalexin, cephradine, fexofenadine) including the mother, if breastfeeding the patient.
16. Use of the following medications within 90 days prior to enrollment: riluzole, valproic acid, hydroxyurea, sodium phenylbutyrate, butyrate derivatives, creatine, carnitine, growth hormone, anabolic steroids, probenecid, agents anticipated to increase or decrease muscle strength, agents with known or presumed HDAC inhibitory effect, and medications with known phototoxicity liabilities (e.g., oral retinoids including over-the-counter formulations, amiodarone, phenothiazines and chronic use of minocycline; patients who are on inhaled corticosteroids, administered either through a nebulizer or an inhaler, will be allowed in the study).
17. Recently initiated treatment for SMA (within 6 weeks prior to enrollment) with oral salbutamol or another β2-adrenergic agonist taken orally is not allowed. Patients who have been on oral salbutamol (or another β2-adrenergic agonist) for ≥6 weeks before enrollment and have shown good tolerance are allowed. The dose of β2-adrenergic agonist should remain stable as much as possible for the duration of the study. Use of inhaled β2-adrenergic agonists (e.g., for the treatment of asthma) is allowed.
18. Any prior use of chloroquine, hydroxychloroquine, retigabine, vigabatrin or thioridazine, is not allowed. Use of other medications known to or suspected of causing retinal toxicity within 1 year prior to enrollment is not allowed.
19. Clinically significant abnormalities in laboratory test results, e.g., ALT values exceeding 1.5-fold the upper limit of normal, unless the elevated ALT level is considered of muscular origin (i.e., in the absence of other evidence of liver disease) which is supported by elevated creatine kinase and LDH. Out of range creatine kinase levels should be reviewed in light of the underlying SMA pathology of the patient; elevated levels per se do not disqualify the patient from the study. In the case of uncertain or questionable results, tests performed during screening may be repeated before enrollment to confirm eligibility.
20. Donation or loss of blood ≥10% of blood volume within 3 months prior to screening.
21. Ascertained or presumptive hypersensitivity (e.g., anaphylactic reaction) to RO7034067 or to the constituents of its formulation (see RO7034067 Investigator’s Brochure).
22. Concomitant disease or condition that could interfere with, or treatment of which might interfere with, the conduct of the study, or that would, in the opinion of the investigator, pose an unacceptable risk to the patient in this study.
23. Recent history (less than 1 year) of ophthalmologic diseases (e.g., glaucoma not controlled by treatment, central serous retinopathy, inflammatory/infectious retinitis unless clearly inactive, retinal detachment, retinal surgery, intraocular trauma, retinal dystrophy or degeneration, optic neuropathy, or optic neuritis) that would interfere with the conduct of the study as assessed by an ophthalmologist. Any other abnormalities detected at screening (e.g., retinal layer abnormalities, edema, cystic or atrophic changes) should be discussed with the investigator, the ophthalmologist, and with the sponsor, who will jointly make the decision if the patient may be enrolled in the study. Patients in whom SD-OCT measurement of sufficient quality cannot be obtained at screening will not be enrolled.
24. Any prior use of an inhibitor or inducer of FMO1 or FMO3 taken within 2 weeks (or within five elimination half-lives, whichever is longer) prior to dosing.
